# Contrast-enhanced microCT evaluation of degeneration following partial and full width injuries to the mouse lumbar intervertebral disc

**DOI:** 10.1038/s41598-022-19487-9

**Published:** 2022-09-16

**Authors:** Remy E. Walk, Hong Joo Moon, Simon Y. Tang, Munish C. Gupta

**Affiliations:** 1 Department of Biomedical Engineering, Washington University in St. Louis, St. Louis, MO USA; 2 Department of Orthopaedic Surgery, Washington University in St. Louis, St. Louis, MO USA; 3 Department of Neurosurgery, Korea University College of Medicine, Seoul, South Korea

**Keywords:** Biomedical engineering, Preclinical research

## Abstract

A targeted injury to the mouse intervertebral disc (IVD) is often used to recapitulate the degenerative cascade of the human pathology. Since injuries can vary in magnitude and localization, it is critical to examine the effects of different injuries on IVD degeneration. We thus evaluated the degenerative progression resulting from either a partial- or full-width injury to the mouse lumbar IVD using contrast-enhanced micro-computed tomography and histological analyses. A lateral-retroperitoneal surgical approach was used to access the lumbar IVD, and the injuries to the IVD were produced by either incising one side of the annulus fibrosus or puncturing both sides of the annulus fibrosus. Female C57BL/6J mice of 3–4 months age were used in this study. They were divided into three groups to undergo partial-width, full-width, or sham injuries. The L5/6 and L6/S1 lumbar IVDs were surgically exposed, and then the L6/S1 IVDs were injured using either a surgical scalpel (partial-width) or a 33G needle (full-width), with the L5/6 serving as an internal control. These animals recovered and then euthanized at either 2-, 4-, or 8-weeks after surgery for evaluation. The IVDs were assessed for degeneration using contrast-enhanced microCT (CEµCT) and histological analysis. The high-resolution 3D CEµCT evaluation of the IVD confirmed that the respective injuries were localized within one side of the annulus fibrosus or spanned the full width of the IVD. The full-width injury caused significant deteriorations in the nucleus pulposus, annulus fibrous and at the interfaces after 2 weeks, which was sustained through the 8 weeks, while the partial width injury caused localized disruptions that remained limited to the annulus fibrosus. The use of CEµCT revealed distinct IVD degeneration profiles resulting from partial- and full-width injuries. The partial width injury may serve as an alternative model for IVD degeneration resulting from localized annulus fibrosus injuries.

## Introduction

Mouse models are often used for the preclinical validation of treatments and therapeutic candidates. Mice offer particular advantages of easy maintenance, year-round breeding, short gestation period, large litters, and inbred tolerance. Furthermore, the ability to manipulate the mouse genome enables mechanistic studies with greater biological precision than larger mammalian models. Moreover, the mouse lumbar intervertebral disc (IVD) provides close geometric and microstructural semblance to the human lumbar IVD^1,2^. While the mouse lumbar IVD exhibits age-related degeneration^3,4^, an injury is often utilized to reproduce the inflammatory conditions and accelerate the IVD’s degenerative cascade^5,6^.

To create a targeted injury to the IVD, the surgical exposure of the IVD is required. These commonly involve the posterior-lateral, transperitoneal, and anterior/lateral retroperitoneal surgical access. Of these, the retroperitoneal approach is particularly advantageous in that it: (1) does minimal damage to major organs, vessels, and musculature; (2) accesses multiple spinal levels with a relatively small incision; and (3) enables direct visualization of the target tissue. Moss et al. demonstrated the efficacy and reproducibility of the retroperitoneal approach in rabbits^7^. Exposing the lumbar IVD enables the ability to create a targeted, directed injury for the investigation of the subsequent degenerative process^8–10^. Masuda et al. and Sobajima et al. described the rabbit annulus fibrosus injury model, where the injury was confined to the annulus fibrosus, that caused slow progressive degeneration of the IVD over 8 weeks^11,12^. The phenotype and the progressive nature of this model recapitulates the human disease and may be better suited to explore therapies that leverage prevention or regeneration. In contrast, more damaging approaches that injures both the annulus fibrosus and nucleus pulposus produce rapid and severe course of degeneration^7,13,14^. Ohnishi et al. showed that a puncture injury to the mouse lumbar spine exhibits measurable degeneration as early as 2 weeks^13^. There are several studies describing IVD degeneration using the lumbosacral IVD injury in the mouse model^13,15,16^. In the mouse IVD where the average disc height is approximately 300–400 μm, an injury by needle puncture produces damage to 55–90% across the height and 15–40% across the width of the IVD^2,14^, representing significant trauma that is atypical in human IVDs. Moreover, these procedures involve damage and injury to the nucleus pulposus, such as with a complete puncture to the IVD, with no possibility to decouple the contributions by the annulus fibrosus and the nucleus pulposus toward the ensuing degenerative cascade. Piazza and coauthors have shown that unilateral (half-width) and bilateral (full-width) injuries in the tail IVD results in unique degenerative trajectories, but this has not been investigated in the mouse lumbar spine^14^. We thus sought to compare the degenerative profiles of IVDs after partial- and full-width injuries in the mouse lumbar spine. In order to evaluate the identify the site of injury and degeneration of the IVD in a spatially robust manner, we utilized contrast-enhanced microCT^17,18^, in addition to histological grading, to quantify the changes in structure and composition at 2-, 4- and 8-weeks after surgery.

## Materials and methods

### Animal preparation

All animal procedures were performed with Washington University School of Medicine IACUC approval, with all methods approved by the relevant regulatory entities. Female C57BL/6J mice of 3–4 months age were used (BW: 20–25 g). They were housed under standard animal husbandry conditions (in a temperature-controlled [21 ± 1 °C] room with normal 12-h light/dark cycles).

The partial-width injury mimics a localized injury to the annulus fibrosus, aka annular tear injury, in humans^19^. Whether partial-width injury is associated with IVD degeneration in humans has not been resolved^20,21^. We first evaluated the feasibility of the partial-width injury on six animals, which were euthanized shortly after recovery from anesthesia. These IVDs were harvested, and then the thickness of the annulus fibrosus and the depth of the injury were measured using contrast-enhanced microCT (CEµCT) and histological analyses.

A second cohort of animals were randomly divided into three groups at the time of surgery: partial-width (PW) injury (n = 18), full-width (FW) injury (n = 18), and Sham (n = 15) with all animals undergoing to retroperitoneal surgical exposure of the lumbar IVD to compare the progression of the IVD response following injury. Sample sizes were determined from the preliminary studies with histological scoring as the primary outcome. These groups were allowed to recover after surgery and then cross-sectionally evaluated at 2-, 4-, and 8-weeks post-surgery (n = 5/sham and n = 6/injury). The injury was delivered to the L6/S1 IVD with the L5/6 IVD used as the internal control. The tissues were evaluated using CEµCT and histological analyses for degeneration. All experiments align with the ethical guidelines set out by ARRIVE.

### Retroperitoneal approach to the intervertebral disc procedure

**Figure 1:**
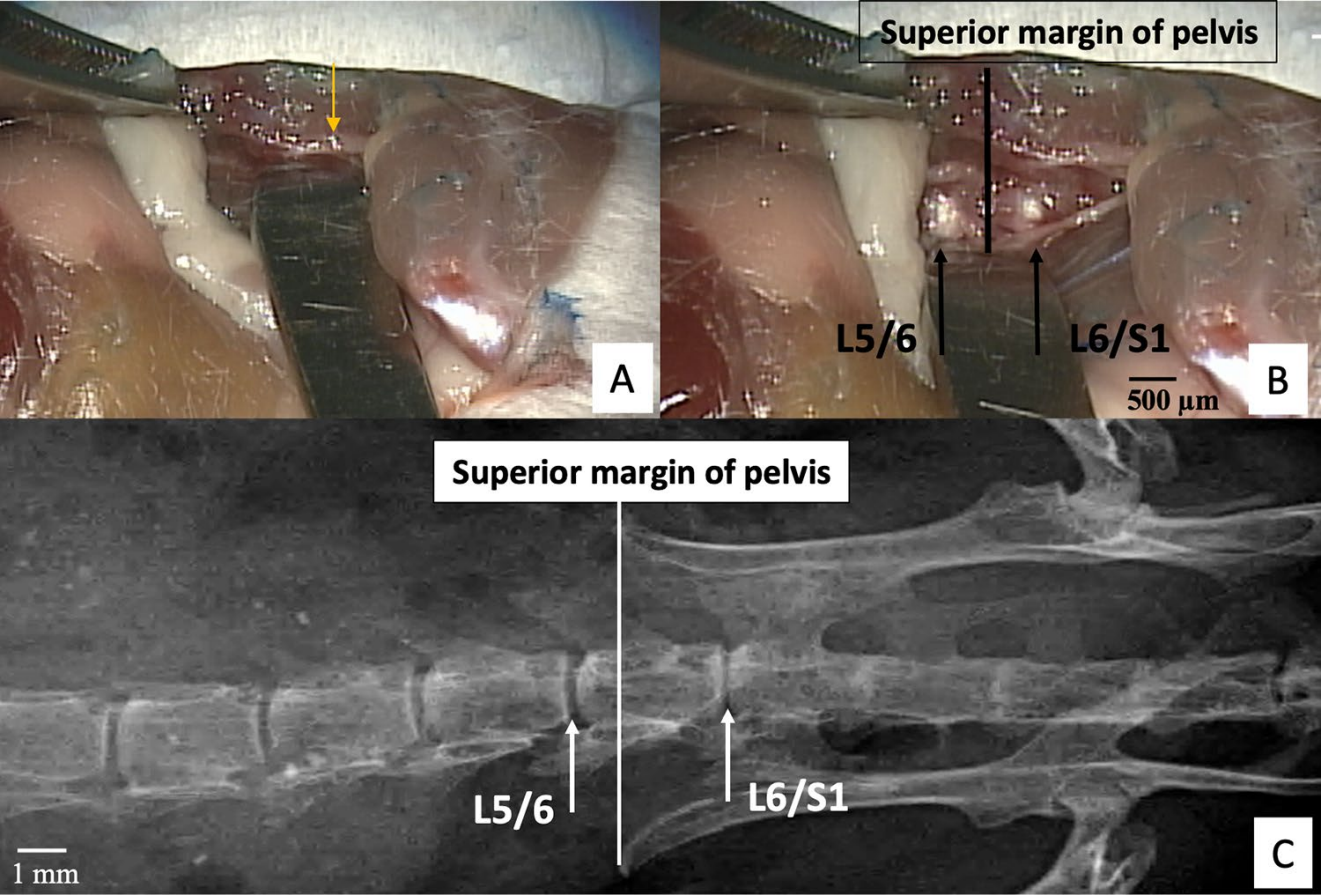
Retroperitoneal dissection exposes the lumbosacral intervertebral disc using microscopic guidance. (**A**) The yellow arrow indicates the left pelvic bone and forceps grip the gluteus muscles. As an optional step, the surgical window can be enlarged further by first holding the iliac crest to keep the pelvis steady, and then abducting and externally rotating the hip by moving the distal femur (also see Fig. S4). The abdominal wall and peritoneum are the retracted anteriorly by a Penfield dissector to expose the psoas muscles. (**B**) The psoas muscle can be stripped posteriorly-to-anteriorly by displacing and retracting the muscles attached to the anterior surface of the pelvis. Superior margin of the pelvic bone indicating the L6 vertebral body can be identified by tactile confirmation of the Penfield dissector, and then L5/6 and L6/S1 IVDs becomes visually evident. (**C**) X-ray confirms the position of the pelvis relative to the L5/6 and L6/S1 intervertebral discs.

Mice were anesthetized with isoflurane gas in oxygen via a facemask (3–4% induction and 2–2.5% maintenance at 1 L/min flow rate; Highland Medical Equipment) and were given a preoperative intradermal injection of lidocaine (7 mg/kg; Hospira, Inc). The left flank was then shaved from the ventral to the dorsal midlines, and the skin was sterilized. The skin was prepared for aseptic surgery via washing with 70% ethanol and povidone iodine. Under microscopic guidance, retroperitoneal dissection was used to expose the lateral aspect of the spine with a Penfield dissector. The pelvis and hip could be rotated posteriorly to expose a broader working space while holding the pelvic bone and distal femur (Fig. S4). The Penfield dissector was used to tug on the peritoneal wall to expose the psoas muscle and to protect the abdominal organs (Fig. 1A). The psoas muscle was retracted posteriorly to anteriorly by initially displacing the tissues attached to the anterior surface of the pelvis using a cotton swab, and then held in place using a metal spatula (Fig. 1A,B). The spinal column and the intervertebral disc were exposed from the ventral midline to the posterior margin of the disc at the L5/6 and L6/S1 levels (Fig. 1B). Since the superior margin of the pelvic bone indicates the L6 vertebral body (Fig. 1E), the location of the L5/6 and L6/S1 IVD could be confirmed (Fig. 1B,C). Extension to the cranial space beyond the L5/6 was also easily achieved by blunt dissection.

All mice were monitored every 8–12 h for 4 days following surgery to monitor activity levels and wound recovery as appropriate. Of 58 mice that underwent surgery, one mouse was euthanized 2 days post surgery due to wound dehiscence, and this animal was replaced in the subsequent surgical cohort. For pain management, mice were given IP injection of carprofen (5 mg/kg; Zoetis, Inc) every 8–12 h supplemented with carprofen tablets (2 mg/tablet; Bio-Serv). Additional details and images on the surgical procedure can be found in Supplemental methods, Supplemental Fig. S1 and on the microCT analysis in Supplemental Fig. S2.

### Partial-width and full-width injuries

For the partial-width injury, a distance of 0.3 mm from the end of the No. 11-scalpel blade tip was measured and marked with a micro-caliper under a microscope (Fig. 3A). The distance of 0.3 mm was determined from our preliminary studies. The edge of the blade was allowed to insert into the IVD until the 0.3 mm marking was no longer visible under the microscope. The injury site was closely observed under the microscope to confirm that there is no leakage of the NP.

For the full-width injury, a 33G needle was inserted through the IVD lateral axis of the IVD and allowed to penetrate both sides of the annulus fibrosus (e.g. ‘bilateral’)^6,8,10,14–16,22,29^ (Fig. S3). In contrast to the partial-width injury, NP herniations were observed following the full-width injury.

### Contrast-enhanced microCT tomography (CEμCT)

Three to four samples from each group were included for contrast-enhanced microCT analysis. Each functional spine unit was incubated in a solution of 175 mg/mL solution diluted from a stock of Ioversol (OptiRay 350; Guerbet, St. Louis) in PBS at 37 °C. After 24 h of incubation, samples were scanned using a µCT40 (Scanco Medical, CH) at a 10-µm voxel size (45 kVp, 177 uA, high resolution, 300 ms integration). Ioversol is a hydrophilic contrast agent allowing for the visualization of the AF and NP on microCT, and its measurements have been shown to correlate with water and glycosaminoglycan contents^17,22^.

CEµCT data was exported as a DICOM file for analysis in a custom MATLAB program (https://github.com/WashUMRC/ContouringGUI). After an initial median filter (sigma = 0.8, support = 5), the functional spine units were isolated from surrounding soft tissue not part of the IVD by drawing a contour around the outer edge every 5 transverse slices and morphing using linear interpolation. The IVD was manually segmented from the vertebral bodies with the same methodology as above. The remaining voxels were designated as the whole disc mask. The NP was thresholded from the AF followed by a morphological close and then a morphological open to first fill interior holes and then smooth the boundaries. The volumes and average attenuations (intensity) were calculated from the regions within the masks of the NP and whole disc. The volume was determined from the total number of voxels contained within the mask, and the attenuation was taken as the average 16-bit grayscale value of the voxels. Visualizations of the microCT were obtained using the image processing application OsiriX (Pixmeo, Geneva). AF thickness, partial-width injury depth and disc height index (DHI) were measured along the mid-sagittal plane. DHI was calculated as the ratio of the IVD height to width. IVD height was taken as the average at 5 equidistant points along the mid-sagittal plane (Fig. S2A). The ratio of NP intensity/disc intensity (NI/DI), defined as the average attenuation of voxels in the NP mask divided by the full disc mask (Fig. S2B), is an fully three-dimensional measure that quantifies the relative size and hydration to inform the relative changes in degeneration^23^. Evaluation was randomized and evaluator was blinded to the group of the sample while processing data.

### Histological preparation and evaluation

Following microCT, samples were fixed for 24 h in 10% neutral buffered formalin followed by 3 days of decalcification in Immunocal (StatLab 1414-X). The samples were embedded in paraffin, sectioned at a thickness of 10 µm, and then stained with Safranin-O and Fast Green. The histological classification system recently developed by Melgoza et al. in 2021 was used to quantify the degeneration of the injured level (L6/S1) which allows the quantification of changes in multiple IVD compartments nucleus pulposus, annulus fibrosus, endplates and interface boundaries^24^. Histological grading was done blinded. Morphology and NI/DI determined from CEµCT was used to further inform the level of degeneration of the injured level (L6/S1) compared to the internal control (L5/6).

### Measuring thickness of AF and the depth of partial-width injury

The CEμCT of the injured L6/S1 and histological analysis on uninjured L5/6 from the first cohort of mice were used to measure the thickness of the AF. The CEμCT on the injured L6/S1 was used to measure the depth of injury as defined by the shortest perpendicular distance from the outer edge of annulus fibrosus to the visually observable outline of the injury site.

### Statistics

All statistics were examined for normality and nonparametric tests were used accordingly. A 2-way ANOVA was used to assess the effects of injury and postsurgical time on histological grade with adjusted post hoc comparisons between groups (Tukey). Paired *t* tests were used to compare the contrast-enhanced microCT parameters of the injured IVD with the uninjured adjacent IVD in the same animal. Comparisons and effects were considered significant when the p-value is less than or equal to 0.05. All statistics were run using GraphPad Prism 9.3.1 (GraphPad, San Diego, California).

## Results

A total of 58 mice were included in the study. The average surgery time was 15 min 38 s ± 6 min 23 s from incision to closure. There was no mortality or complications due to the surgery or the immediate recovery. The animals were observed to recover full ambulation with no notable limping or paralysis. Caution was taken to prevent injury of the lumbosacral plexus located posteriorly during the blunt dissection of the psoas muscle from posterior to the anterior direction.

### Annulus fibrosus (AF) thickness

**Figure 2:**
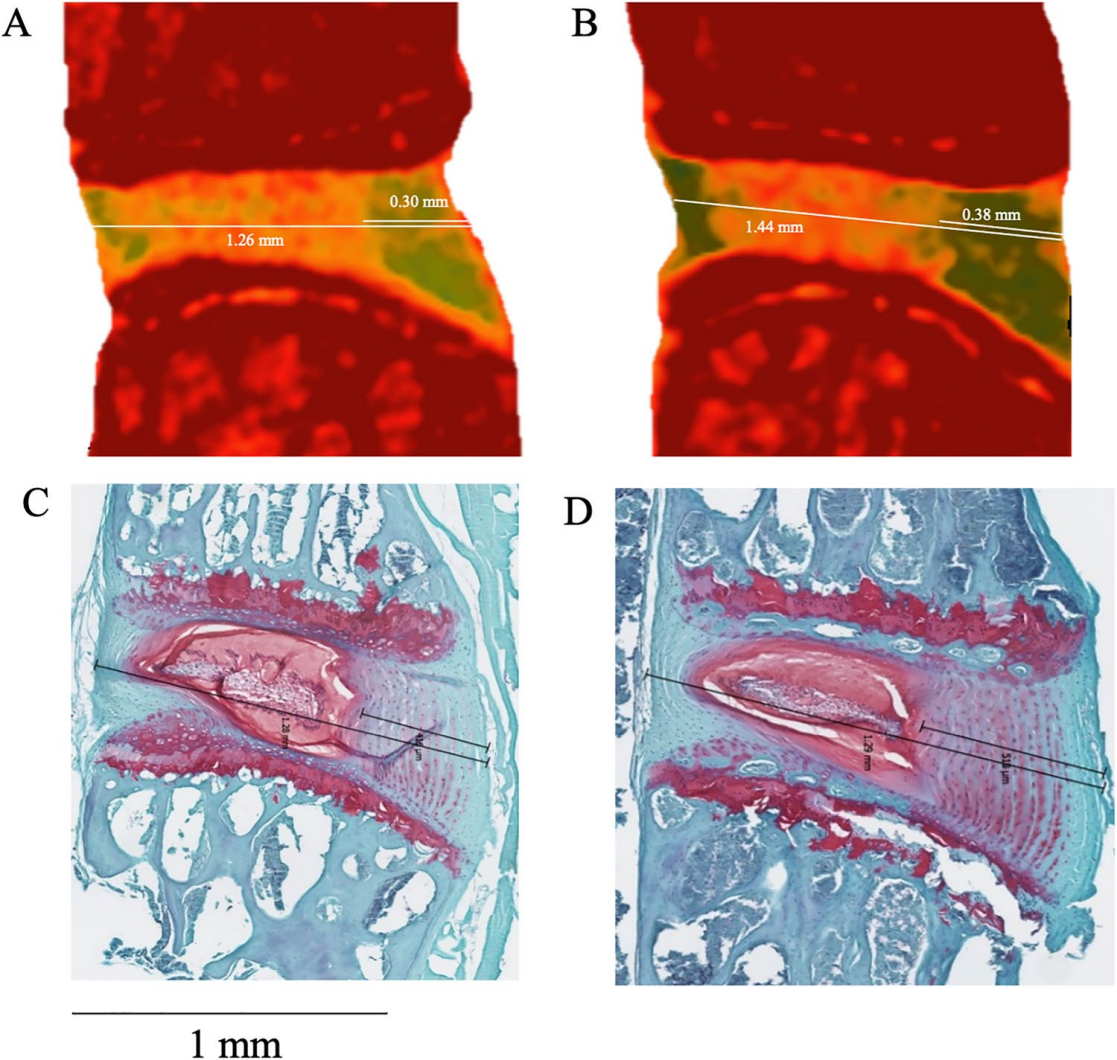
Measuring the thickness of the intervertebral disc and the annulus fibrosus. (**A**,**B**) Contrast-enhanced microCT (CEμCT) from the IVD adjacent to the partial-width injury, immediately following surgery. (**C**,**D**) Histology were used to measure the IVD widths and anterior AF thicknesses. The CEμCT and histology measurements were highly correlated (r^2^ = 0.96, p < 0.001) and statistically indistinguishable (paired *t* test, p = 0.37), confirming the fidelity of the non-destructive evaluation of CEμCT.

CEμCT and histology measurements of AF thickness and IVD width were highly concordant. The anteroposterior IVD width measured with CEμCT was 1.27 ± 0.13 mm (mean ± standard deviation), whereas that measured by histological analysis was 1.21 ± 0.11 mm. AF thickness measured with CEμCT was 0.38 ± 0.05 mm and that measured with histological analysis was 0.43 ± 0.10 mm (Fig. 2). The CEμCT measured values were statistically indistinguishable from (p = 0.37) and were highly correlated (r^2^ = 0.96) with the histologically measured values (Fig. 2).

### Depth of partial-width injury

**Figure 3:**
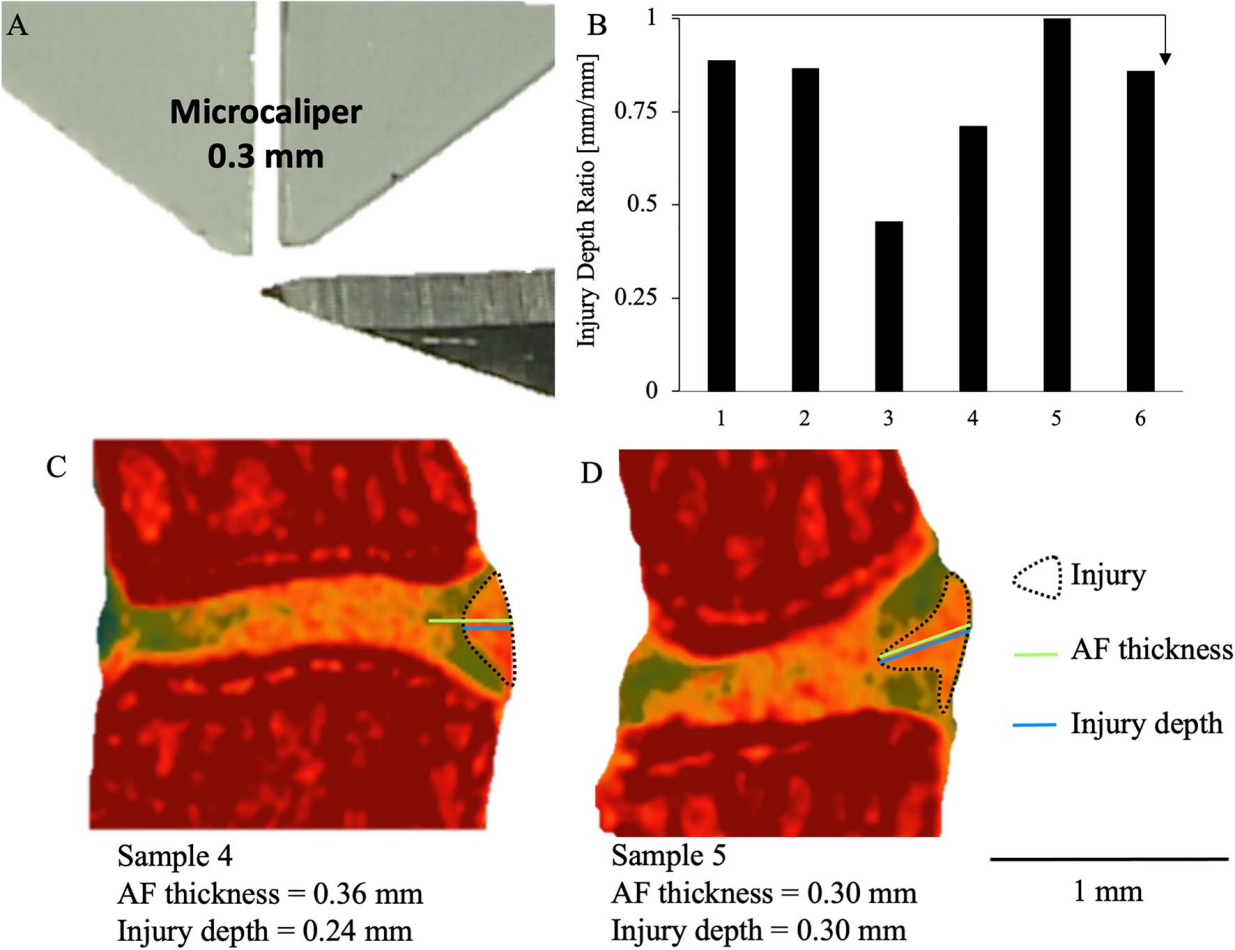
Depth of the partial-width injury measured by contrast-enhanced micro-computed tomography. (**A**) The tip of the scalpel edge is marked at 0.3 mm to allow for visual confirmation during surgery that the partial-width injury is limited to the AF. These mice were euthanized after recovering from anesthesia immediately following PW injury, (**B**) All of the injury depths were confirmed by CEµCT and histology to have depths that do not exceed of the AF in each injured IVD, confirmed by the ratio of injury depth to AF thickness which no greater than 1 in all samples. (**C**,**D**) Representative CEµCT showing the range of depths achieved by this AF injury.

The ratio of the injury depth to the AF thickness was 0.80 ± 0.19 (Fig. 3B). In sample 5, although the depth of the injury approached the width of the annulus fibrosus, no herniations was observed. (Fig. 3C). The depth of injury measured with CEμCT was 0.29 ± 0.05 mm. (Fig. 3B).

### Structural evaluation of the IVD

**Figure 4:**
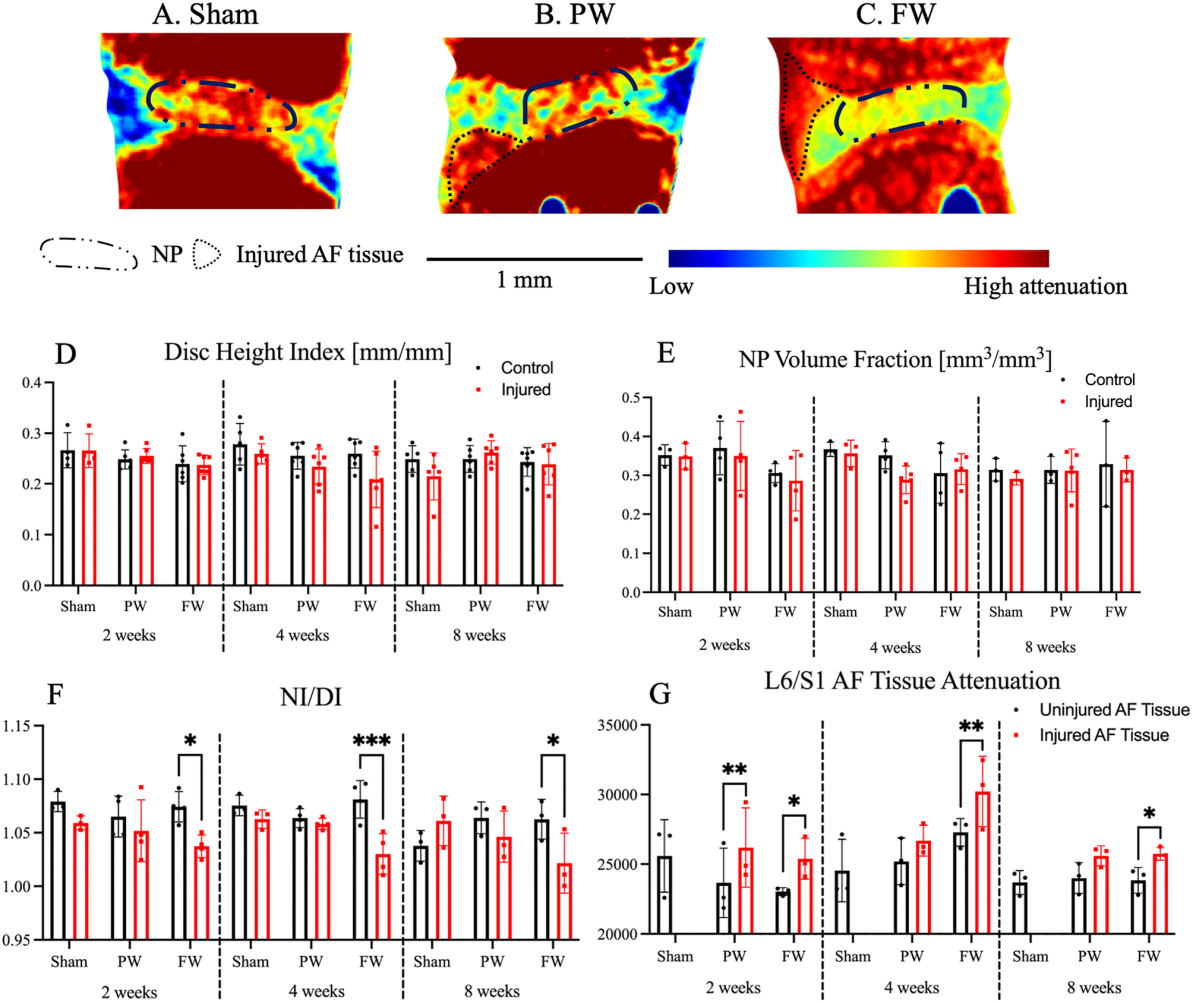
Full-width (FW) injury induces a sustained loss of NP hydration compared to both the partial-width (PW) injury and Sham groups. (**A**–**C**) Contrast-enhanced microCT of injured levels (L6/S1) at 2 weeks following injury in both FW and PW groups reveals the site of injury. Scale bar is 1 mm. CEµCT analyses were done on n = 3–4/group. No changes were observed in the whole IVD structure, as evidenced by **(D**) disc height index (DHI) and (**E**) NP volume fraction, computed between injured (FW and PW) and uninjured levels. (**F**) NI/DI of injured level (L6/S1) and uninjured control level (L5/6). The ratio of the nucleus pulposus intensity to the whole disc intensity of the FW injured IVDs at all timepoints revealed the loss hydration and proteoglycans in the nucleus pulposus that is indicative of degeneration (NI/DI—approximately 4% drop in intensity). (**G**) FW injured IVDs consistently have higher AF attenuation around the site of injury, while the PW injured IVDs exhibit an initial increase in the AF attenuation that recovers over time (n = 3/group).

Neither the partial-width nor the full-width injuries resulted in changes in the Disc Height Index across the 2-, 4-, and 8-week time points (Fig. 4D). Similarly, the size of the nucleus pulposus, characterized by the nucleus pulposus (NP) volume fraction, were not dramatically different between groups and time points (Fig. 4E). The NI/DI revealed that the full-width injury caused a dramatic decrease in the relative attenuation of the NP, indicating a loss of NP hydration, at the 2-, 4-, and 8-week time points (Fig. 4F). The impact of the partial- and full-width injuries to the annulus fibrosus was observable at 2 weeks. The attenuation of the annulus fibrosus (AF) increases around the site of injury, and this is evident in both the partial- and full-width groups. Two weeks after injury, both the partial- and full-injuries showed an increase attenuation of the injured AF tissue, with the full-width injury group sustaining this increase (Fig. 4G).

### Extent of IVD degeneration

**Figure 5:**
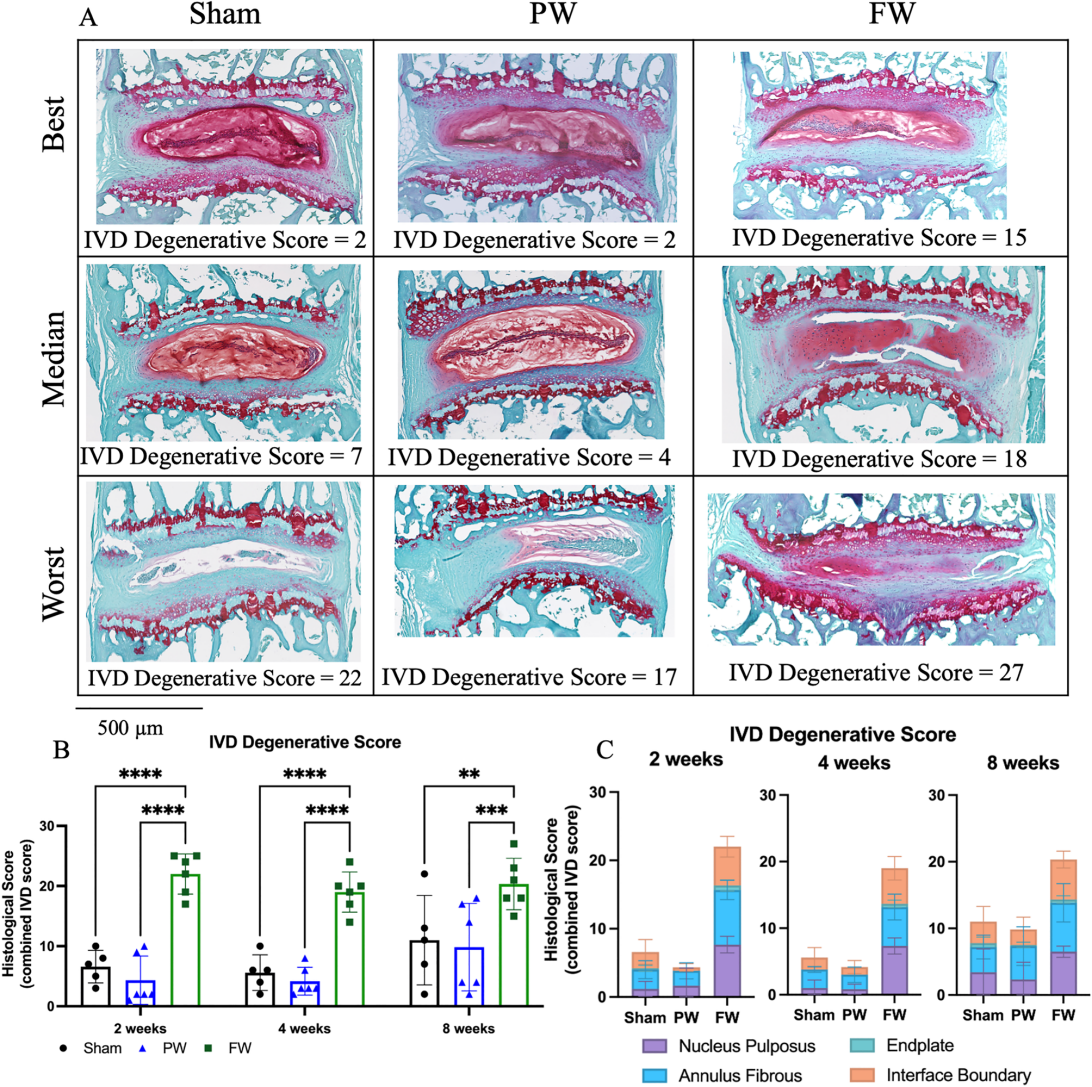
FW induces rapid and sustained IVD degeneration compared to both Sham and PW. (**A**) Safranin-O stained histological section of injured level (L6/S1) (n = 6/injured and 5/sham for each timepoint). The histological images in each group, combined by time-points, were ranked by combined histological score and the lowest (Best), median, and highest (Worst) are shown to provide the full spectra of observed degeneration. Scale bar equals 500 µm. (**B**) Total IVD degenerative scores of L6/S1 for all groups. (**C**) Breakdown of NP, AF, endplate and interface boundary contributions to the combined IVD degenerative score. Significant degeneration was observed at all timepoints indicated by significant increases of degenerative scores in NP, AF, interface boundary and combined IVD degenerative grades in the FW group compared to Sham and PW (2-way ANOVA; p < 0.05). While PW causes localized disruption to the AF, it does not appear to cause degeneration.

Quantitative histological analyses revealed a degenerative cascade to the IVD following FW injury as early as 2 weeks post injury but not with the PW injury. The histological classification showed significant degeneration following FW injury at all timepoints (p < 0.05) in the NP, AF, interface boundaries, and total IVD degenerative score (but not the endplate score) while no differences were detected between PW (Fig. 5). No morphological differences between timepoints or injury groups were observed (Fig. 4D,E).

## Discussion

Animal models of IVD degeneration are imperative to elucidate the key molecular mechanisms of the pathophysiology. To date, rodents, rabbits, dogs, goats, sheep, and primates have been used as models for intervertebral disc degeneration^11^. One main advantage of mouse models is the availability of reagents and modifications that could be used concomitantly with surgically induced IVD degeneration. However, the small size of the mouse requires a high degree of surgical precision, particularly with access and exposure of the lumbar spine. While many studies utilize the mouse tail for degenerative models^12,15,25,26^, the lumbar spine maintains the anatomical proximity to physiologically relevant structures such as the dorsal root ganglions.

We validate here a novel procedure for mouse lumbosacral IVD injury with visual guidance via microscopy and gross features. The retroperitoneal space of the spine can be located (Fig. S1) with the left pelvic bone with gluteus muscles and proximal thigh as landmarks (Fig. 1A,B). Blunt-dissection of the fat pad surrounding the thoracic cage anteriorly exposes the thigh muscles posteriorly and the gluteus muscles superiorly as landmarks (Fig. 1A). The lateral approach to the IVD allows for precise injury to the annulus fibrosus of the IVD (Fig. 1). Exposure of the pelvic bone from the gluteus muscle and rotation of the pelvic bone posteriorly is necessary to achieve exposure of multiple IVDs in the surgical field. The psoas muscles can be easily displaced and retracted posterior-anteriorly with a cotton swab and a metal spatula. CEµCT allowed for a 3D spatially robust quantification of morphology and composition^23^ that confirmed the localization of injury across the cross-section time points. CEµCT measurements of the IVD morphology were in excellent agreement with the histological measures. NI/DI and histological analyses indicated degeneration of injured IVDs starting 2 weeks post-surgery.

The present study compared the post-surgical responses between a localized experimental injury with AF-limited depth and a more commonly used needle puncture injury. The technique described herein also focused on achieving a consistent depth and size of injury. The marked tip of the No. 11-scalpel blade provided guidance for the AF-limited injury and resulted in no observed NP leakage. While most injury models cause damage to the nucleus pulposus, the cartilaginous endplates, and even the vertebral body^27–29^, the partial-width injury here results in an isolated injury to the outer annulus fibrosus. Surprisingly, we observed no degenerative changes in the endplate in the full-width injury, suggesting that it is possible to induce an IVD only injury using a carefully applied 33G needle. Following full-width injury, IVDs exhibited rapid degeneration as early as 2 weeks after injury, and this is sustained through the 4- and 8-week time points. It is worth noting that the Sham IVDs also exhibited some degree of degeneration. Although these IVDs were not injured using a needle, the animals endured surgical trauma to expose the IVD that likely produced elevated levels of cytokines. Prior work show that sustained exposure to inflammatory conditions can cause IVD degeneration^6^, and that is likely the case here. Despite maintaining disc height, the FW injury caused consistent degenerative changes in the nucleus pulposus, annulus fibrosus, and interface boundaries which included the AF-endplate and NP-AF boundaries. The PW injury did not produce statistically significant degenerative changes, but whether there were innervation or vascularization changes at the outer annulus fibrosus remains to be investigated. The mouse lumbar IVD injury model, while useful, has some limitations. Mouse IVDs present mild to moderate degeneration even with severe injuries. Relative to the size of the animal, the surgical exposure of the IVD creates substantial trauma for the animal and may have contributed to inflammation-induced degeneration in some sham IVDs. In the context of the current study, eight weeks may not be long enough to observe the long-term degenerative response due to the injury. Nevertheless, the ability to utilize animal models creates an opportunity to investigate and modulate the biological processes associated with IVD degeneration.

CEµCT allowed for nondestructive visualization of the injury site following both partial- and full-width injuries. Compared to high field MRI which can resolve features on the order of 200 × 200 × 200 μm^3^, the CEµCT technique used here was conducted at a resolution of 10 × 10 × 10 μm^3^, which represents an 8000-fold improvement in resolution^17^. The increased attenuation on CEµCT indicates changes in composition and hydration resulting from the disruption in the annulus fibrosus with both injuries (Figs. 3, 4). The staining of the water-rich compartments by Ioversol is reversible and compatible with downstream histological analyses.

While the full-width injury to the nucleus pulposus induces a more rapid and severe course of degeneration that is more aligned with an acute trauma^7,11,12^, the partial-width model may enable mechanistic investigations of how the IVD responds to injuries of the annulus fibrosus without IVD degeneration. Consistent with unilateral injuries of the tail IVD, the resultant degenerative cascade was nuanced^14^, and required high resolutions modalities to detect measurable changes. The PW injury may be more useful for studying the acute IVD injury response independently of IVD degeneration.

## Supplementary Information

Supplementary Information.

Supplementary Figures.

## Data Availability

The datasets used and/or analyzed during the current study available from the corresponding author on reasonable request.
