# Supplementary material for: Contrast-enhanced microCT evaluation of degeneration following partial and full width injuries to the mouse lumbar intervertebral disc
Source: Sci Rep. 2022 Sep 16;12:15555. doi: 10.1038/s41598-022-19487-9 (PMC9481554; doi:10.1038/s41598-022-19487-9)
Supplement: Supplementary file 1 — Supplementary Information. [file 41598_2022_19487_MOESM1_ESM.docx]

**Title:** Contrast-enhanced microCT evaluation of degeneration following partial and full width injuries to the mouse lumbar intervertebral disc

**Authors:** +Remy E. Walk, MS^1,2^; +Hong Joo Moon, MD, PhD^2,3^; Simon Y. Tang, PhD, MSCI^1,2*^; Munish C. Gupta, MD^2^

**Supplemental Methods**

***Instrumentation***

Instruments used in surgery included a size No. 11-scalpel blade (Henry Schein Medical**)**, forceps (Fisher Scientific**)**, dissecting scissors (VWR**)**, Penfield dissector (VWR**)**, q-tips (Dukal Corporation**)**, 4-0 nylon sutures (Ethicon**),** suture grabbers (Fisher Scientific**),** and microscope (Zeiss**).**

***Methodology***

*Retroperitoneal approach to the intervertebral disc procedure:* The left flank was shaved from the ventral to the dorsal midlines using veterinary trimmers. Mice were then transferred to the operating suite and positioned in the left lateral decubitus position. The skin was prepared for aseptic surgery via washing/rinsing with povidone iodine scrub and alcohol rinse. The animals were draped to isolate the prepped area. Isoflurane by inhalation was used to maintain surgical depth anesthesia throughout the procedure.

There were two surface landmarks utilized for this approach: 1) the anterior margin of the thigh (vertical) and bony prominence of the pelvis (horizontal) and 2) the fat pad reflected underneath the skin. The fat pad located between the anterior margin of the thigh and abdominal wall was utilized as an access point to the retroperitoneal space. A 1–1.5-cm horizontal incision was made along the bony prominence of the pelvic bone line covering the entry and the anterior 1/3 of the thigh (Figure S1A). The vertical caudal margin of the access point was retracted and resected by blunt dissection with Mosquito forceps (Figure S1B and S1C). The gluteus muscles and left pelvic bone (a) were utilized as a grip to hold and move the spine axis with forceps. The opening to the retroperitoneal space (c) between (a) and oblique abdominal muscles (b) was carefully blunt-dissected until the psoas muscle was exposed (Figure S1D).
