# Supplementary material for: Contrast-enhanced microCT evaluation of degeneration following partial and full width injuries to the mouse lumbar intervertebral disc
Source: Sci Rep. 2022 Sep 16;12:15555. doi: 10.1038/s41598-022-19487-9 (PMC9481554; doi:10.1038/s41598-022-19487-9)
Supplement: Supplementary file 2 — Supplementary Figures. [file 41598_2022_19487_MOESM2_ESM.docx]

**Title:** Contrast-enhanced microCT evaluation of degeneration following partial and full width injuries to the mouse lumbar intervertebral disc

**Authors:** +Remy E. Walk, MS^1,2^; +Hong Joo Moon, MD, PhD^2,3^; Simon Y. Tang, PhD, MSCI^1,2*^; Munish C. Gupta, MD^2^

**Supplemental Figures**

**
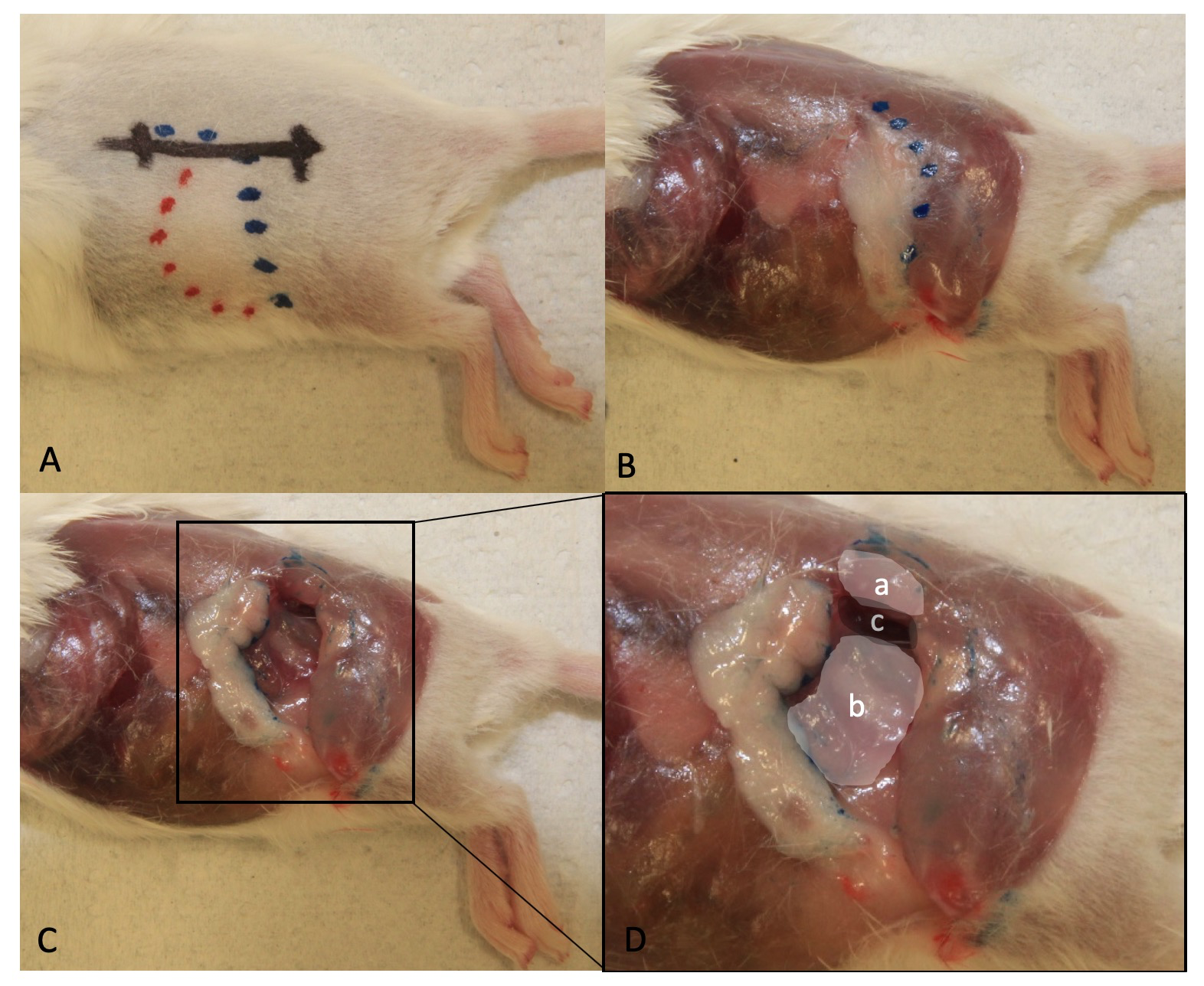
Supplemental Figure 1.** *Lateral retroperitoneal approach to the mouse lumbosacral spine* **(A):** The superficial anatomical landmarks are denoted by 1) the blue dotted line which outlines anterior margin of the thigh and bony prominence of the pelvis, and 2) the red dotted line which outlines the fat pad located between the thigh and abdominal wall. The black solid line is an approximately 1 cm incision along the pelvis line that reveals the surgical access point adjacent to the anterior thigh. (**B and C):** The access point to the retroperitoneal space lies between the blue and red dotted lines. The underlying fat pad and anterior margin of the thigh can be easily separated and retracted to expose the retroperitoneal space by blunt dissection. (**D):** The pelvis is rotated posteriorly to expose a broad working space by gripping (a) with forceps. The area (c) between (a) and (b) is carefully separated by blunt dissection to expose the psoas muscle: (a) gluteus muscles and left pelvic bone; (b) oblique abdominal muscles; (c) opening to the retroperitoneal space.


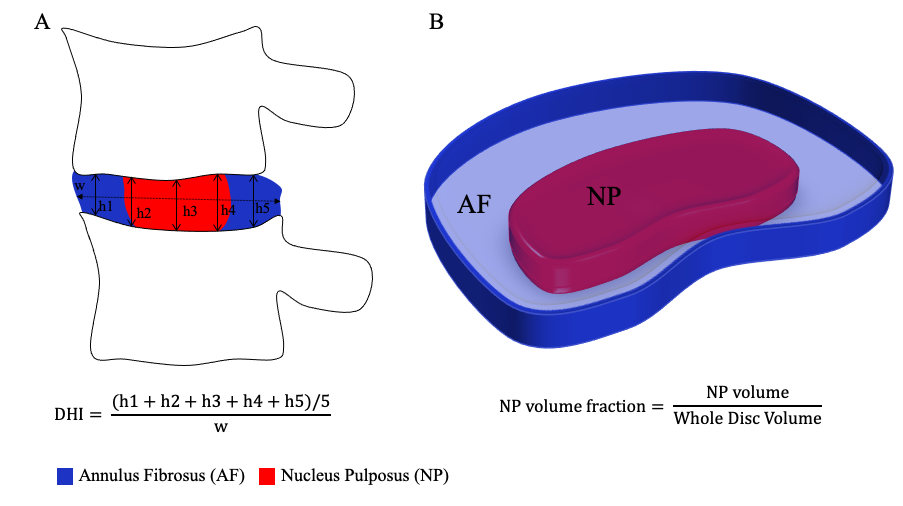


**Supplemental Figure 2.** *Contrast-enhanced microCT morphological analyses.* (A) Disc height index (DHI) is the ratio of the average IVD height to IVD width at a mid-sagittal section. IVD height is measured at five equidistant locations across the entire width of the IVD. The whole IVD is manually contoured, and the NP is thresholded out. (B) NP volume fraction is taken as the ratio of the NP volume to whole disc volume. AF volume is the whole disc volume minus NP volume.

*
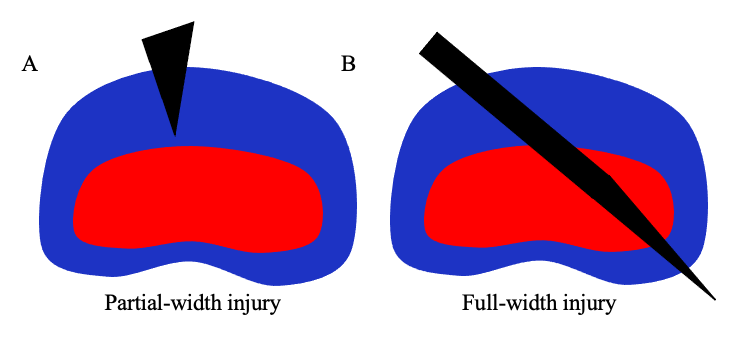
*

**Supplemental Figure 3.** *Representative figures of injury models.* (A) Partial-width injury is isolated to the annulus fibrous on the anterior edge of the IVD. The max width of the scalpel that enters the IVD is estimated at 0.5 mm (approximately the IVD height at the anterior edge) compared to the 0.21 mm diameters of the 33G needle used in (B) full-width injury.


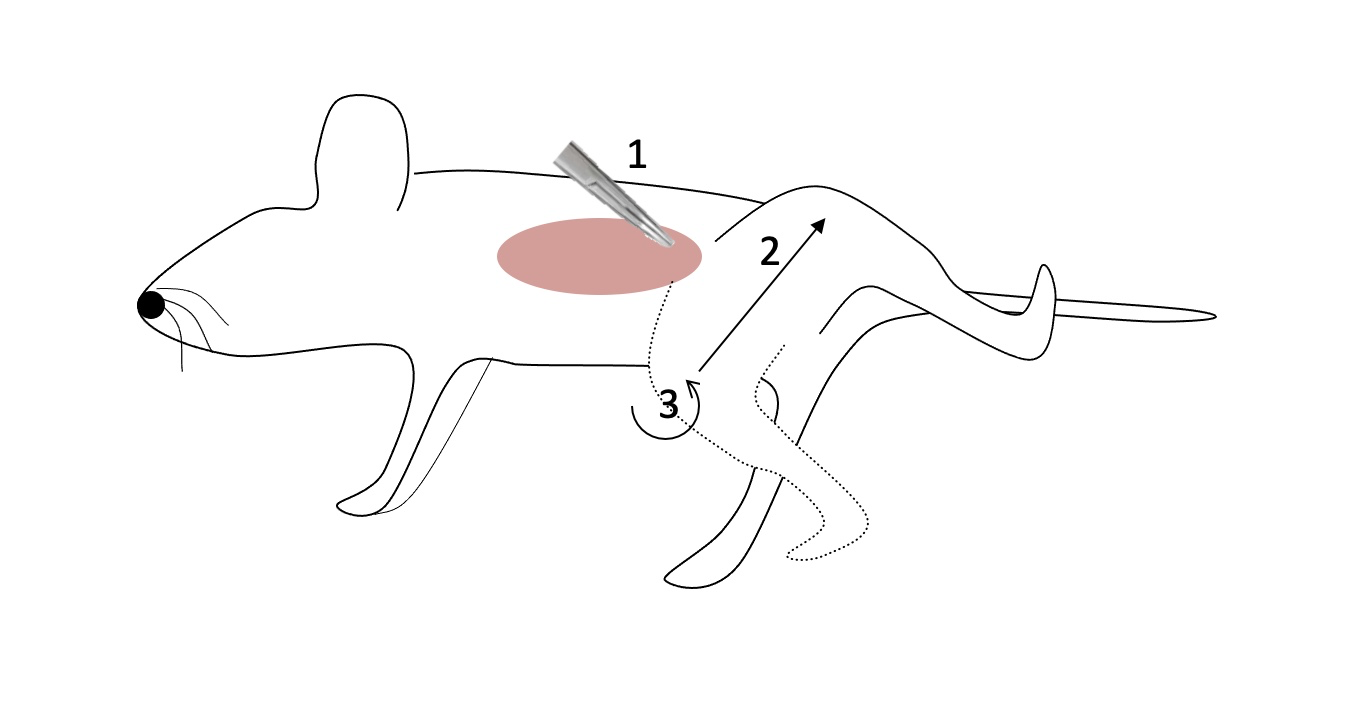


**Supplemental Figure 4.** *Schematic for enlarging the surgical window to the lower lumbar IVDs through abduction and externally rotating the hip.* (1) First, the iliac crest is held using forceps to keep the pelvis steady. (2) The hip is then abducted and (3) externally rotated by moving the distal femur. These optional motions increases the working space immediately surrounding the surgical window and minimizes the tissue contraction during the procedure.
